# Time series classification of multi-channel nerve cuff recordings using deep learning

**DOI:** 10.1371/journal.pone.0299271

**Published:** 2024-03-12

**Authors:** Aseem Partap Singh Gill, Jose Zariffa

**Affiliations:** 1 Institute of Biomedical Engineering, University of Toronto, Toronto, ON, Canada; 2 Department of Chemical Engineering and Applied Chemistry, University of Toronto, Toronto, ON, Canada; 3 KITE—Toronto Rehabilitation Institute—University Health Network, Toronto, ON, Canada; 4 Rehabilitation Sciences Institute, University of Toronto, Toronto, ON, Canada; 5 Edward S. Rogers Sr. Department of Electrical and Computer Engineering, University of Toronto, Toronto, ON, Canada; Air University, PAKISTAN

## Abstract

Neurostimulation and neural recording are crucial to develop neuroprostheses that can restore function to individuals living with disabilities. While neurostimulation has been successfully translated into clinical use for several applications, it remains challenging to robustly collect and interpret neural recordings, especially for chronic applications. Nerve cuff electrodes offer a viable option for recording nerve signals, with long-term implantation success. However, nerve cuff electrodes’ signals have low signal-to-noise ratios, resulting in reduced selectivity between neural pathways. The objective of this study was to determine whether deep learning techniques, specifically networks tailored for time series applications, can increase the recording selectivity achievable using multi-contact nerve cuff electrodes. We compared several neural network architectures, the impact and trade-off of window length on classification performance, and the benefit of data augmentation. Evaluation was carried out using a previously collected dataset of 56-channel nerve cuff recordings from the sciatic nerve of Long-Evans rats, which included afferent signals evoked using three types of mechanical stimuli. Through this study, the best model achieved an accuracy of 0.936 ± 0.084 and an F_1_-score of 0.917 ± 0.103, using 50 ms windows of data and an augmented training set. These results demonstrate the effectiveness of applying CNNs designed for time-series data to peripheral nerve recordings, and provide insights into the relationship between window duration and classification performance in this application.

## 1. Introduction

Neurological injuries and amputation may cause significant loss of motor function, resulting in deterioration of the quality of life for affected individuals [1, 2]. Neuroprosthetics and neuromodulation devices seek to restore the functionality of movements and improve the quality of life for affected individuals. This is achieved through stimulation of the nervous system, known as neurostimulation. Some applications of neurostimulation include the restoring the sense of touch to prosthesis users [3–5] and functional electrical stimulation (FES) for movement restoration [6–8].

To improve the effectiveness of implanted FES systems, it is desirable to perform neurostimulation using an implantable closed-loop control device. A closed-loop system is ideal as it integrates information about the current state of the body and the environment. This allows for closed-loop systems to adapt to unexpected perturbations and produce robust and smooth movements compared to open-loop systems [6, 9, 10]. The sensory information required for closed-loop control is contained in afferent nerves and can be recorded using peripheral nerve interfaces.

Categories of peripheral nerve interfaces include extraneural electrodes, intraneural electrodes, and regenerative electrodes [11, 12]. Increasing the invasiveness of an electrode increases the signal-to-noise ratio (SNR) of the signal and places electrical contacts closer to axons, increasing the functional selectivity of the recordings. However, higher invasiveness entails a greater likelihood of damage to the neural tissue, leading to a potential decrease in stability for long-term implantation, which is a limitation when designing implantable closed-loop control devices. Extraneural electrodes are the least invasive, as they lie outside the nerve, but provide lower SNR and signal selectivity. Increasing the information that can be extracted from extraneural electrodes is a possible avenue towards achieving a better trade-off between invasiveness and selectivity. To this end, multi-channel extraneural recording involves collecting information using multiple extraneural contacts and increases the potential information that can be extracted through the corresponding signals. In particular, deep learning excels at leveraging large amounts of complex and noisy data, and offers an opportunity to obtain more precise and reliable information from peripheral nerve recordings.

Several signal processing algorithms have been applied to analyse and interpret recorded multi-channel peripheral nerve signals [13]. Recently, Koh et al. saw success in applying a 2-dimensional convolutional neural network (2D CNN), named Extraneural Spatiotemporal Compound Action Potential Extraction Technique Network (ESCAPE-NET), to this task [14, 15]. ESCAPE-NET leverages both the spatial arrangement of the electrodes and the temporal nature of the data recorded by each electrode to create a spatiotemporal signature (a 2D matrix with contacts in one dimension and time samples in the other). Arranging the recordings in this fashion allows for the use of 2D CNNs to learn both spatial and temporal relationships in the data, and classify individual naturally evoked compound action potentials (nCAPs) according to the neural pathway where they originated.

While CNNs originally gained widespread application for processing 2D image and video data, specialized architectures have also been proposed that are adapted to time-series data through the use of 1-dimensional kernels [16]. Electroneurography (ENG) is fundamentally time-series data, and therefore an appropriate target for 1D CNN architectures, as well as for transformer models for time-series data. An interesting feature of this type of approach is the ability to integrate various timescales of data. While ESCAPE-NET emphasizes the spatial arrangement of the data, it does not integrate multiple timescales as effectively. This limitation could potentially be addressed through the use of 1D CNN or transformer architectures designed for time series data.

The objective of this study was to leverage the time series nature of multi-channel ENG by implementing, modifying, and evaluating various multi-scale 1D CNN and transformer architectures’ ability to classify peripheral nerve extraneural signals based on the pathway of origin.

## 2 Materials and methodology

### 2.1 Dataset

The dataset used in this study was collected previously by our group. A description of the protocol can be found in [15]. Briefly, ENG signals were collected from the sciatic nerve of Long-Evans rats using a 56-contact spiral polyimide nerve cuff electrode (CorTec GmbH, Freiburg, Germany), 23 mm in length and 1mm in diameter. The nerve cuff configuration consisted of 7 evenly spaced rings each with 8 contacts. Data were acquired using a neural acquisition board (RHD2000, Intan Technologies, USA) at a frequency of 30 kHz. Three types of mechanical stimuli were applied to the rat’s hindpaw: dorsiflexion, plantarflexion, and pricking of the heel using a Von Frey monofilament (300 g). These stimuli evoke afferent activity in the tibial, peroneal, and sural fascicles of the sciatic nerve, respectively, thereby producing different patterns of bioelectric activity at the surface of the nerve [17–19]. Dorsiflexion and plantarflexion signals were recorded by manually dorsiflexing and plantarflexing the rat’s foot at approximately 60° in sync with a metronome set to a tempo of 70 beats per minute. On the first beat, the foot was moved to the dorsiflexion/plantarflexion position, then returned to the neutral position on the second beat. For recording heel prick signals, the Von Frey Monofilament was pricked in tune with a metronome similar to dorsiflexion and plantarflexion. On the first beat, the heel was pricked and held until the second beat. 100 trials of each stimulation were recorded. The stimulus applied served as a ground truth signal for the classifiers, whose task was to associate a window of recorded signal with the correct stimulus type.

The experimental procedures were approved by the Animal Care Committee of the University of Toronto and all experiments were performed in accordance with the Animal Care Committee’s guidelines.

### 2.2 Preprocessing

The raw data were preprocessed off-line in MATLAB to facilitate the detection of nCAPs in the neural recordings. First, a tripole reference was applied to the collected signals, with the average of the two outermost rings serving as the reference. A tripole reference helps to reduce artefacts from bioelectric sources external to the cuff while preserving signals that result from the propagation of action potentials through the nerve [20, 21]. Next bandpass filtering was applied using a 6^th^ order Butterworth filter with a 1–3 kHz passband, to remove signal content unrelated to the neural activity of interest. Following this, a delay-and-add operation was used, following the methodology of velocity selective recording (VSR). VSR allows for the discrimination of CAPs based on both the direction and conduction velocity of the neural signal [22–24]. To this end, averaged recordings from each ring of contacts were shifted by a delay and then summed, resulting in constructive interference when the delay was consistent with the propagation velocity of the nCAPs and the electrode spacing. The delay-and-add operation transformed the multi-channel data into a single signal in which the SNR of the target signals was increased, to facilitate the detection of nCAPs. A threshold was then applied, using the median absolute deviation estimate approach (Eq 1) [25].


$$Threshold=4\times \frac{Median(|X|)}{0.6745}$$ (1)

**Fig 1:**
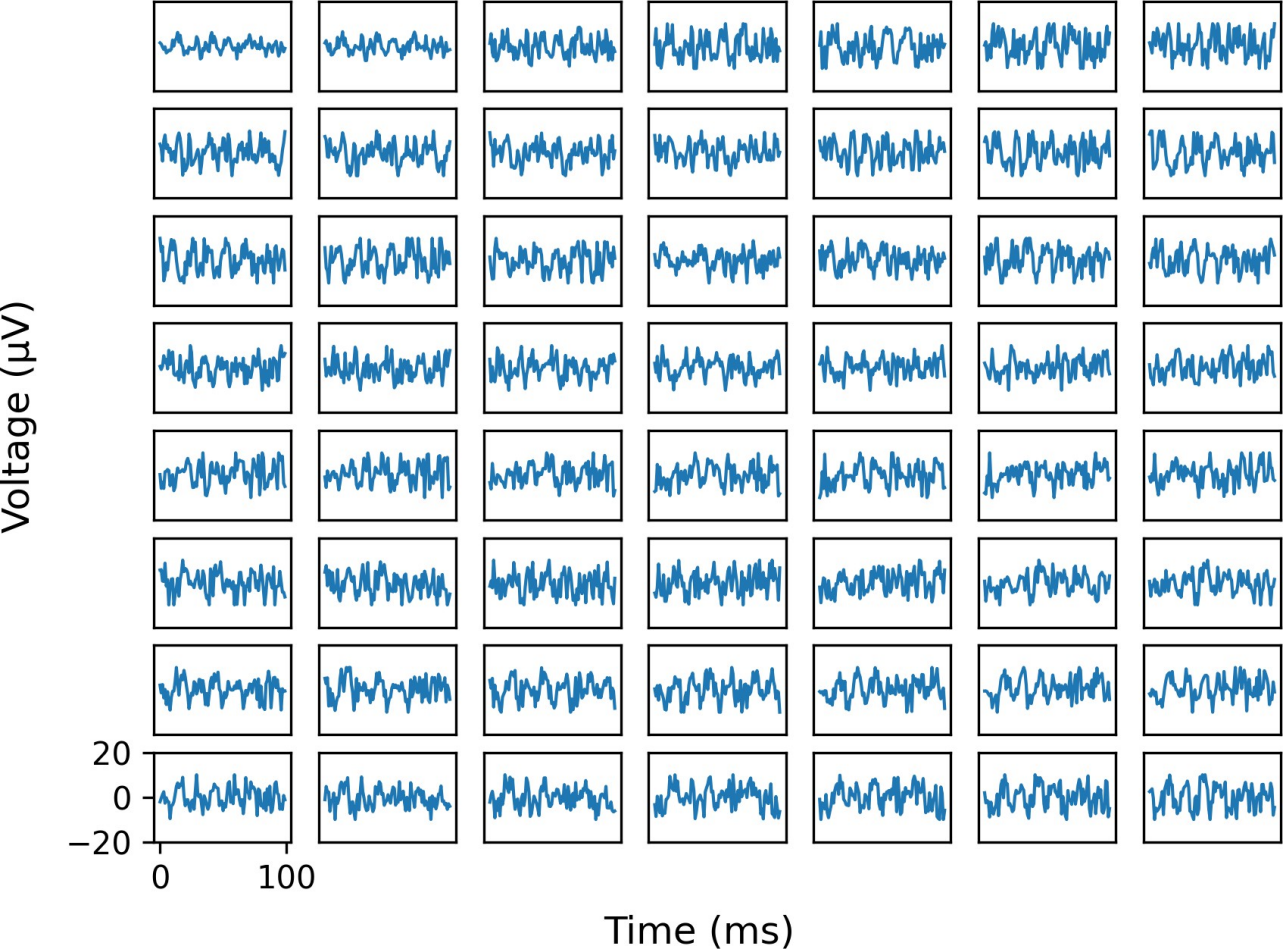
Example of multi-channel ENG data after pre-processing. Columns correspond to different rings of contacts in the nerve cuff electrode, and rows correspond to contacts within a ring.

The delay-and-add step was used only for nCAP detection. Data was then extracted from the pre-processed multi-channel signals for the following steps. The detected nCAP locations were treated as the center of the signal windows to use in the classifier evaluations, with *n* time samples taken after the spike peak and *n*−-1 time samples taken before the spike peak. This yielded signals that were 2n samples long. Three different datasets were prepared, with windows of length 2250 samples, 1500 samples, and 100 samples. The purpose of this comparison was to characterize the relationship between classification accuracy and window length, which has implications for system latency and the likelihood of overlap between the activity of multiple neural pathways. 100 samples is the length of input used by ESCAPE-NET, and therefore enables a direct comparison between architectures. Longer windows may enable time-series models to capture information in longer time scales, at the cost of decreased temporal resolution. A depiction of the processed signal can be found in Fig 1.

Windows larger than 2250 samples were not selected as further increasing window size increases model size, model training time, and the latency of the model.

### 2.3 Data augmentation

**Fig 2:**
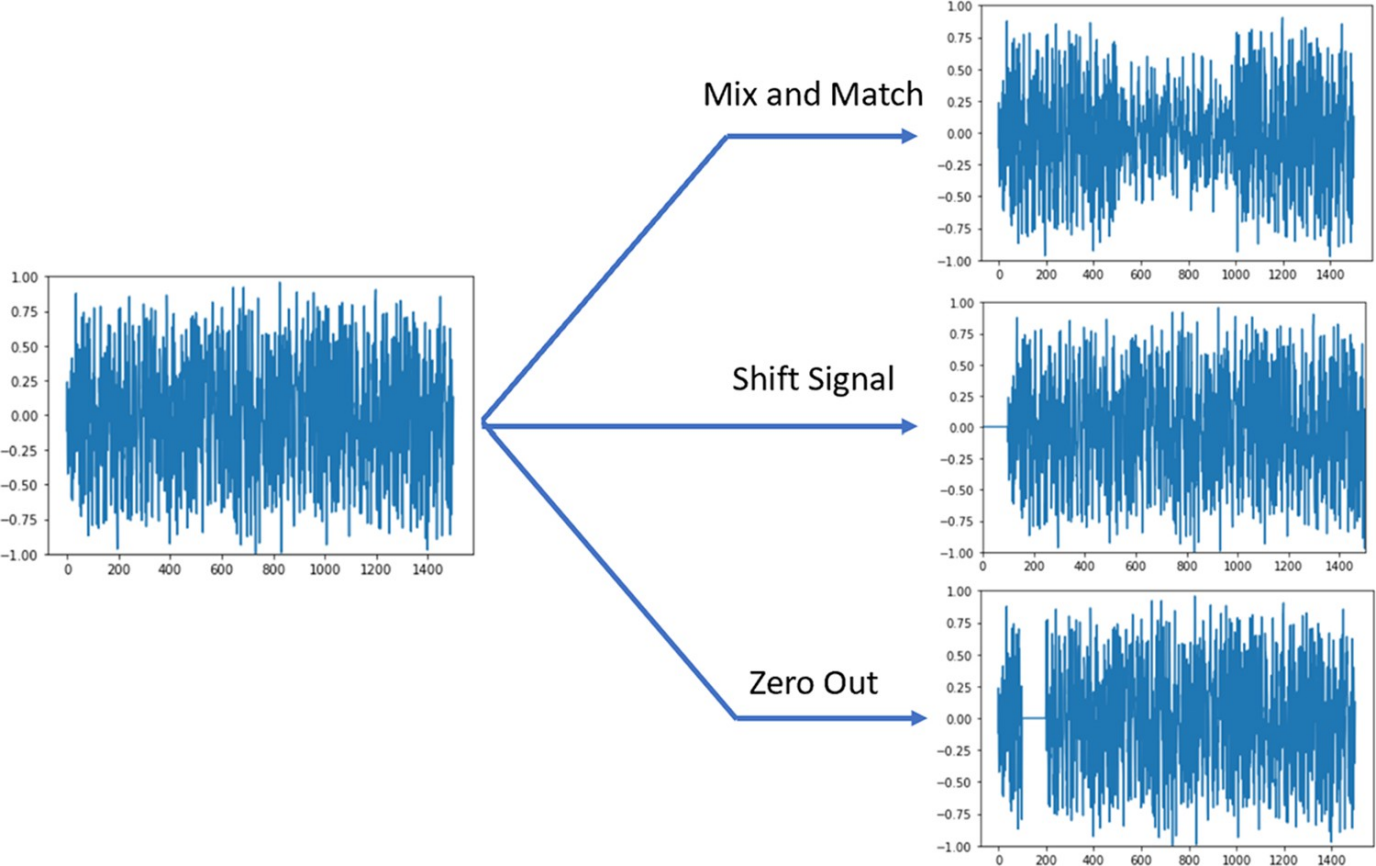
Visualization of the data augmentation techniques applied.

Three types of data augmentation were applied: zeroing out parts of the signals, mixing and matching signals, and shifting signals as shown in Fig 2 [26]. To zero out parts of the signals, up to 10% of the signal was set to zero in either one or two sections. Mixing and matching signals involved taking the middle one third of a signal and replacing it with the middle one third of a different signal of the same class. Shifting the signal was completed by randomly moving the signal up to 5% of the signal’s length forward or backward.

These three methods were selected as they are unlikely to impact the classification of the signal. The main concern was introducing additional spikes, which may potentially occur when using different data augmentation techniques such as jittering, magnitude warping, or flipping [26].

Data augmentation was performed only on the training and validation data, after separating out the testing set for each rat. For the training and validation set of each rat, data augmentation was applied to increase the number of samples per class to 1.5 times the class type with the most sample. This prevented class imbalance from biasing the model’s decisions.

### 2.4 Architectures

Based on literature research, three architectures were selected, optimized, and evaluated. All architectures were implemented in PyTorch. The first architecture is *Multi-Scale Convolution Block (MSCB)* which drew inspiration from Roy [27]. Roy investigated the use of a multi-scale 1D CNN for the classification of electroencephalogram (EEG) motor imagery signals into one of three classes [27]. This network was selected for its high performance, as it achieved 93.4% classification on the motor imagery classification task [27]. Furthermore, the EEG data collected for Roy’s study is collected using three electrodes placed on the head which is similar in structure to the dataset used in this study.

The second architecture was *InceptionTime* [28]. InceptionTime was selected since it was proven to have performance similar to state-of-the-art models on a standardized time-series dataset (UCR), and for its ability to be extended for multi-variate time series [28].

The third architecture was *TARNet* [29], selected for two reasons. Firstly, TARNet saw high performance compared to state-of-the-art models on time series classification and reconstruction tasks. Secondly, TARNet is a transformer model instead of a 1D CNN. Transformers have recently seen state-of-the-art success in various domains including computer vision and text generation [30, 31]. This success if attributable to the low inductive bias of transformer models compared to other deep-learning architectures [32]. However, the drawback of low inductive bias is that transformers require larger amounts of data [33].

The MSCB and InceptionTime models are both 1D CNN models. CNNs have higher inductive bias than transformers as they are built around leveraging the convolutional operator and kernels rather than the attention mechanism [34]. As data is limited in this study, higher inductive bias will likely improve model performance. Furthermore, both CNN models were selected for their use of multiple convolution kernel sizes, which can capture different scales of information in the signals [27, 35, 36].

For all models, kernel sizes were chosen based on an initial exploratory analysis and the parameters provided in their respective papers. In all cases, the exploratory analysis demonstrated that increasing the kernel size would eventually lead to a drop in performance. This is expected since increased kernel sizes will increase the number of parameters in the model. Larger models require more data to train; however the dataset size is fixed in this study.

#### 2.4.1 Multi-Scale convolution block (MSCB)

MSCB architectures were implemented in two versions. The first method used a single classification path, involving only one output. The second method includes the addition of an auxiliary classifier. These models are outlined below.

**Fig 3:**
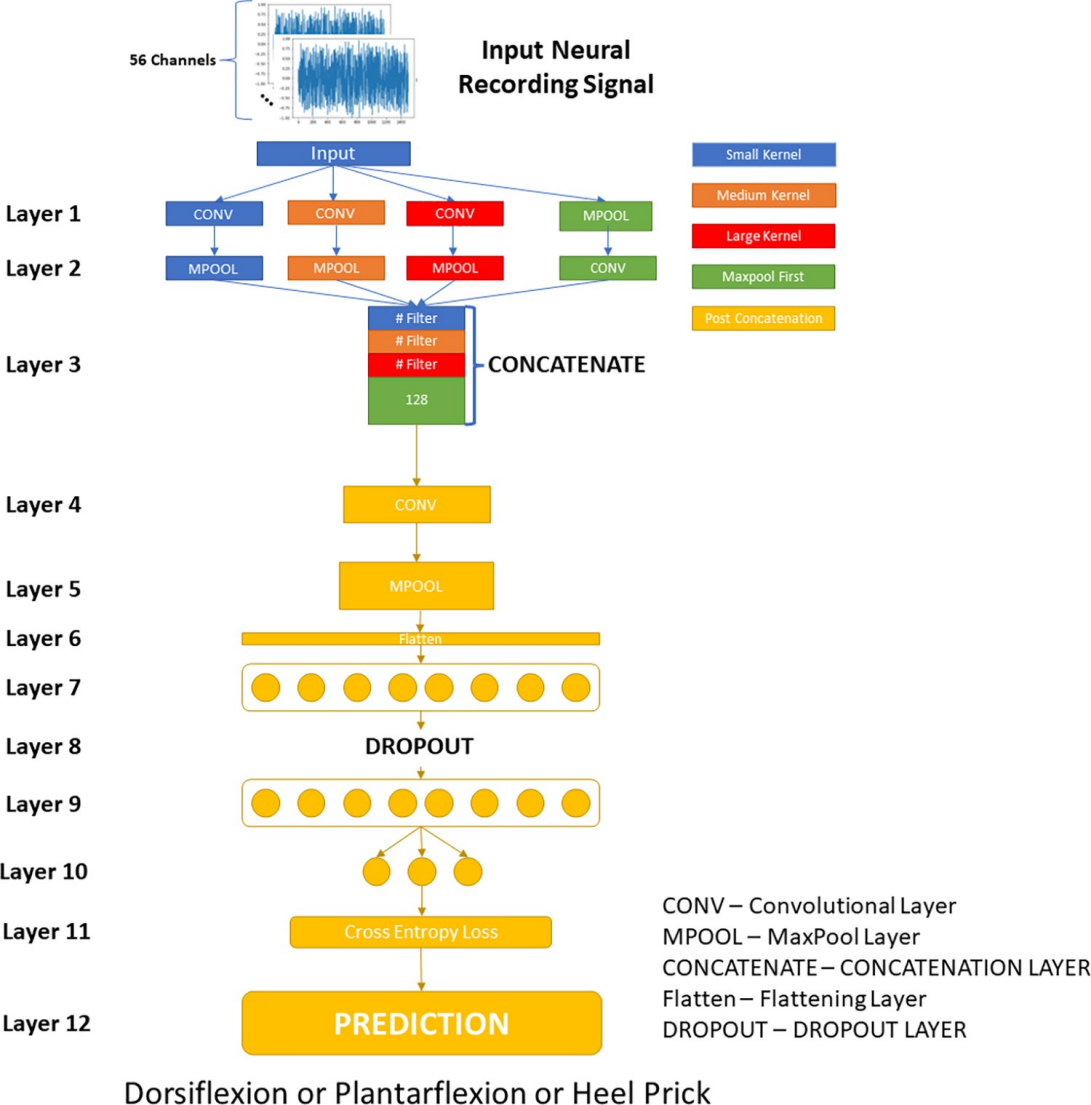
MSCB architecture.

The Single Classifier MSCB architecture network is depicted in Fig 3.

The multi-scale portion of the network occurs from the initial two layers containing four parallel pathways. These pathways are labelled with different colours in Fig 3. The pathways are the small kernel, medium kernel, large kernel, and maxpool pathway. The first three pathways include a convolutional layer followed by a maxpool layer with kernel sizes of 1, 3, and 5 for the small, medium and large pathways respectively. The fourth pathway has a maxpooling (with a kernel size of 3 and a stride of 5) first then a convolution with a kernel size of 24. The outputs of each of these pathways are concatenated along the filter dimension. Following concatenation, the output is fed into a convolutional layer (with a kernel size of 112), followed by a maxpooling layer, and finally into a multi-layer perceptron (MLP) for classification.

**Fig 4:**
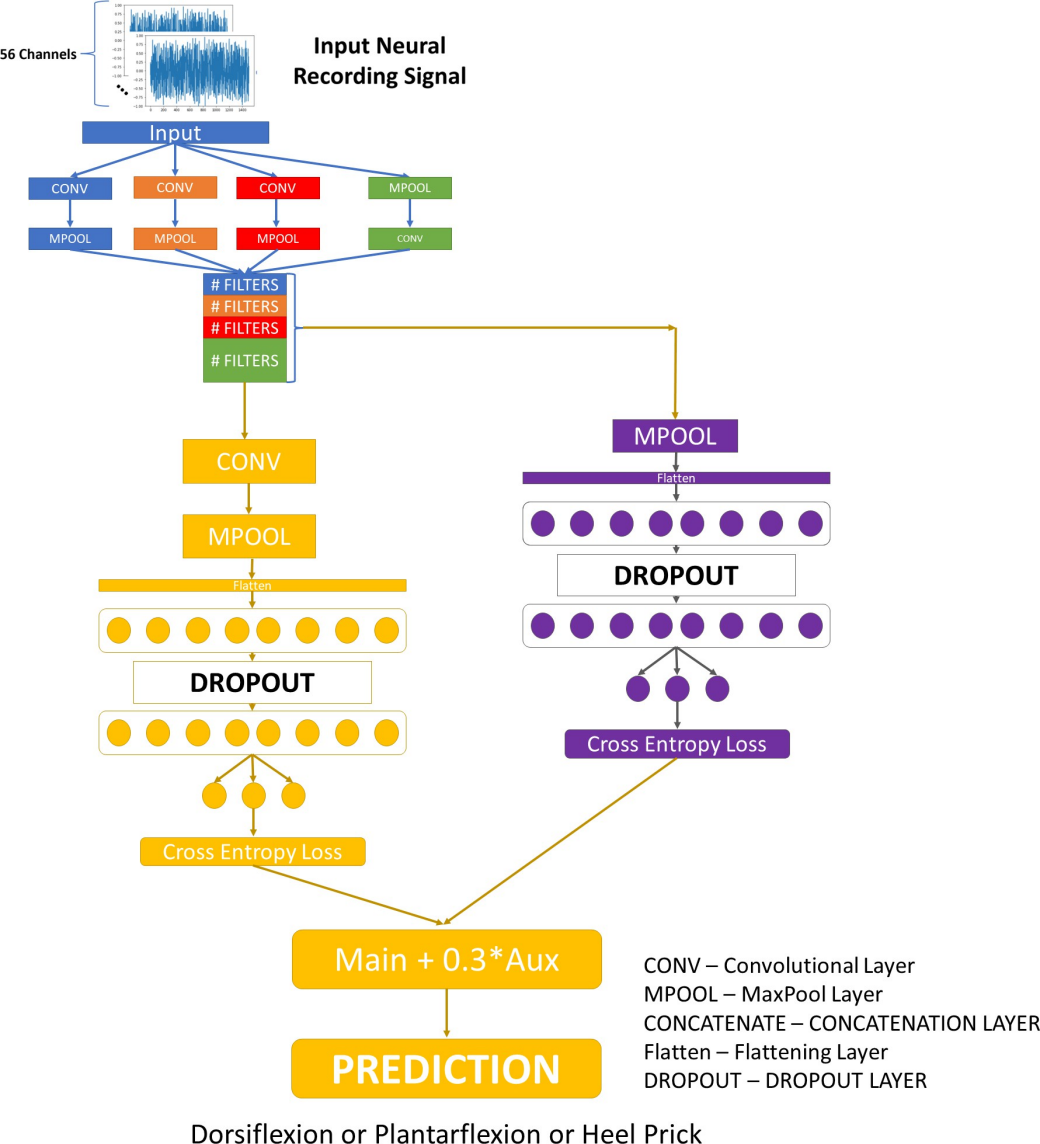
MSCB with auxiliary.

The MSCB Auxiliary network is depicted in Fig 4. The auxiliary pathway (shown in purple) is used to improve model performance by adding a classification with less feature extraction and a smaller receptive field.

The MSCB Auxiliary architecture has the same backbone as the Single Classifier MSCB. The difference is the addition of a second classification pathway following the concatenation layer. This is referred to as the auxiliary classifier of the network. The auxiliary pathway takes the output of the concatenation layer and feeds it into a max pooling layer. Then the pooling layer output is flattened and fed into an MLP. After the MLP layers, the outputs of the main pathway and auxiliary pathway are added together, with the output of the auxiliary pathway multiplied by 0.3.

#### 2.4.2 Inceptiontime

**Fig 5:**
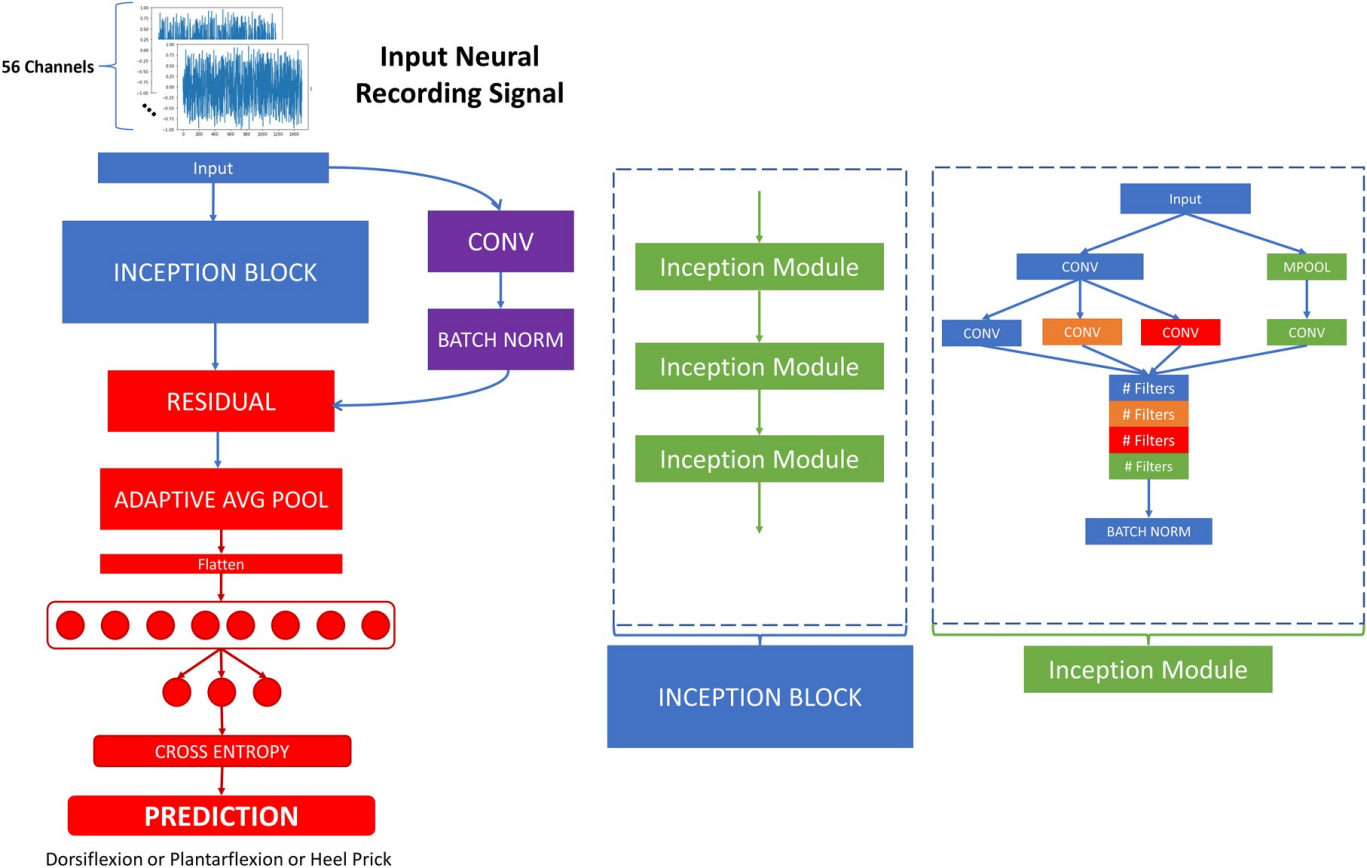
InceptionTime architecture.

InceptionTime sought to draw inspiration from the Inception network by Google for image classification [28]. The InceptionTime architecture is depicted in Fig 5.

The InceptionTime architecture is built up of an Inception block, followed by a residual connection to the input and finally an MLP. The residual connection takes the input and feeds it into a pointwise convolutional layer followed by a batch normalization layer before adding it to the Inception Block output. The Inception Block is made up of three Inception Modules in series. Each Inception Modules starts by feeding the input into two parallel pathways one containing a pointwise convolutional layer and the other containing a maxpooling layer with kernel size of 3. The output of the convolutional layer is fed into another three parallel convolutions each with different kernel sizes (9, 19, and 39), while the output of the maxpooling layer is fed into a single pointwise convolutional layer. All four of these outputs are concatenated together and then fed into a batch normalization layer. After the residual, an average pooling layer is applied, followed by flattening and feeding into an MLP for classification.

InceptionTime is inherently much deeper than MSCB, making it harder to train. However, InceptionTime can easily integrate auxiliary losses by including classifications following one of the earlier parallel convolutions.

#### 2.4.3 TARNet. The architecture of TARNet is depicted in Fig 6

**Fig 6:**
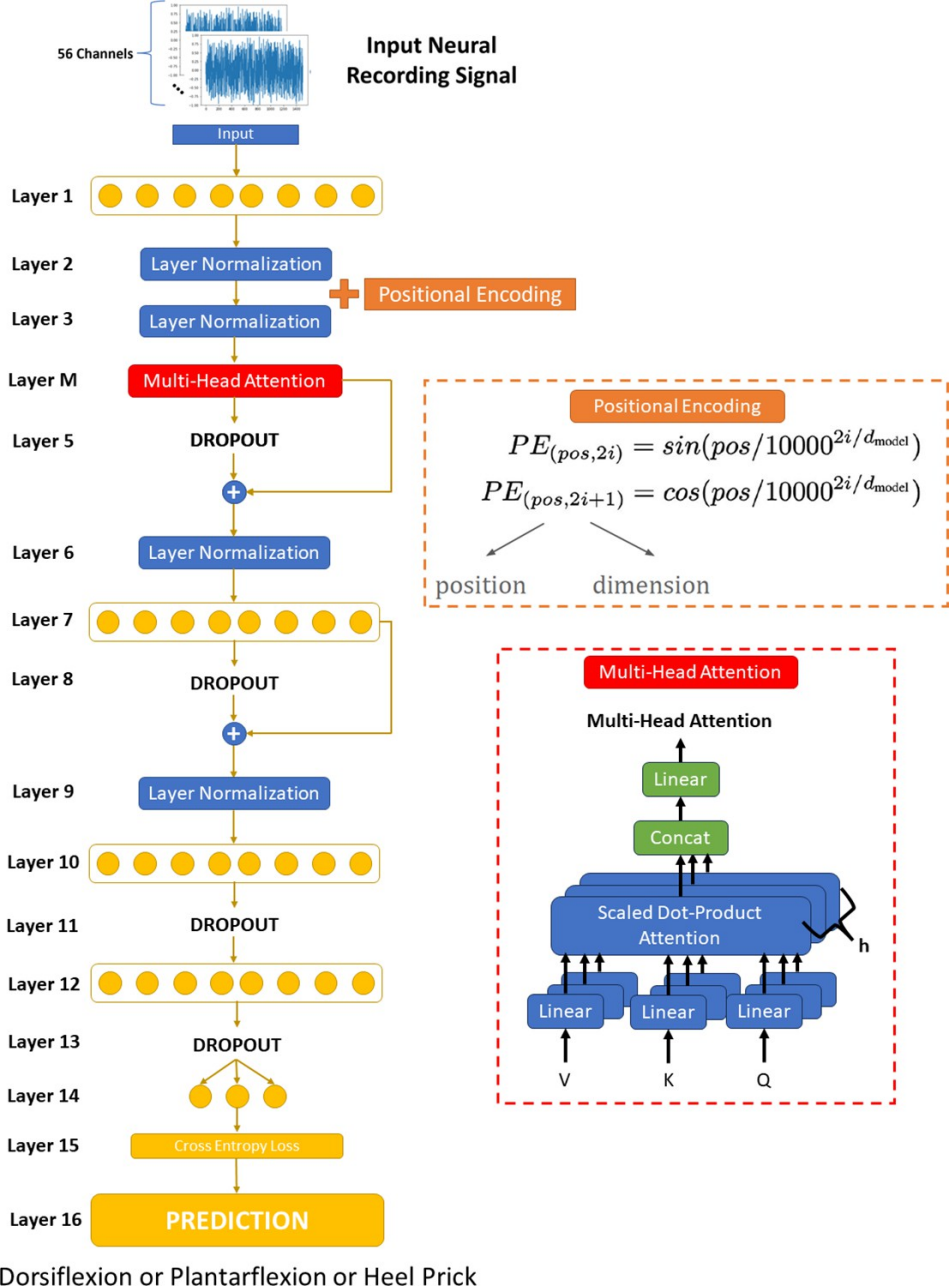
TARNet architecture.

TARNet starts with a linear layer, the purpose of which is to generate the input embedding for the multi-head attention layer. The output of the linear layer is normalized and then combined with a positional encoding. The positional encoding helps the model learn the sequential nature of the data [37]. This is required because without a positional encoding the transformer does not have the inductive bias to infer the significance of the data sequence [37]. In this case, the sequence of the data is significant because the order of the samples in the signal contains crucial information. After adding the positional encoding, the output is normalized once again. Following normalization, the output is fed into a multi-head attention layer. Attention seeks to learn the significance of the relationship of each point in the input data sequence and every other data point. This generates what is known as an attention matrix [37]. After the attention layer, the output is fed into multiple linear layers, dropout with residual connections, and layer normalizations.

### 2.5 Model training and evaluation

The preprocessed dataset was split into a training/validation dataset, to train and optimize the performance of each model, and a testing dataset, to quantify the model’s ability to generalize to data it was not trained on. The split ratio was 90% training/validation and 10% testing. To prevent training biases, each class in the training/validation sets (dorsiflexion, plantarflexion, and pricking of the heel) was made to have an equal number of samples. This process was different for the augmented and unaugmented datasets. For the augmented dataset, data augmentation was used to increase each class to 1.5 times the size of the largest class as outlined in the Data Augmentation section. For the unaugmented dataset, samples were removed from the larger classes to match the number of samples in the smallest class. This prevents the model from achieving good performance by guessing the most common signal class in the dataset. After balancing the classes, the training/validation set was split using a 3-fold cross-validation approach to ensure confidence in model performance for each rat. For each fold, the data was split 66% for training and 33% for validation. The test set was left unmodified to ensure it is most representative of new data that the model has not seen. Each model was trained on the training folds and evaluated on the validation fold; the performance on each of the folds was averaged and the best models were trained on all folds then evaluated on the test set.

Models were trained in PyTorch on an NVIDIA GEFORCE RTX 3090 GPU. All models were trained for a maximum of 400 epochs with an early stopping patience of 3 epochs which is triggered only if validation accuracy exceeded 85% to prevent overfitting of the training set. 400 epochs were selected, as it was observed all models plateaued well before 400 epochs. Early stopping was only used after validation accuracy exceeded 85% as it was observed that the validation accuracy did not increase monotonically in most cases. Cross entropy loss was used as the loss function with Adaptive Momentum Estimation (ADAM) used as the gradient descent optimizer. All other training hyperparameters can be found in S1 File.

To evaluate model performance, two metrics were selected. The first metric selected was test accuracy. This is the accuracy the model achieves on the test set. The second metric chosen was the testing macro F1-score. The F1-score is the harmonic mean of precision and recall. Macro F1-score is used as it weighs all classes equally, allowing it to reflect any biases in the model to guess or not guess a certain class. Both metrics were treated as equally important when comparing models.

## 3. Results

Table 1 presents the results of the MSCB Single Classifier network on datasets with different signal lengths evaluated on the unaugmented dataset.

**Table 1:** Best results for MSCB network trained on datasets with different window lengths.

Number of Samples in Window (Sampled at 30kHz)	Classification Accuracy	F_1_-score
2250	0.871 ± 0.145	0.832 ± 0.160
1500	0.876 ± 0.142	0.838 ± 0.160
100	0.715 ± 0.117	0.700 ± 0.192

Based on these results, the decision was made to use a window length of 1500 for further experimentation. The results of each model’s performance on the 1500 unaugmented dataset are presented in Table 2. For comparison, ESCAPE-NET achieved an accuracy of 0.808 ± 0.104 and F1-score of 0.747 ± 0.153 using windows of 100 samples.

**Table 2:** Best results from each architecture on the 1500 sample unaugmented dataset.

Algorithm	Classification Accuracy	F_1_-score
1500 Window Length Dataset		
MSCB Single Classifier	0.876 ± 0.142	0.838 ± 0.160
MSCB Auxiliary	0.882 ± 0.130	0.846 ± 0.141
TARNet	0.567 ± 0.951	0.537 ± 0.129
InceptionTime	0.729 ± 0.126	0.673 ± 0.138

After optimization on the unaugmented datasets, each model was evaluated on the augmented dataset. The results of those experiments are provided in Table 3.

**Table 3:** Best results from each architecture on the 1500 timepoint augmented dataset.

Algorithm	Classification Accuracy	F_1_-score
1500 Signal Length Dataset		
MSCB Single Classifier	0.936 ± 0.084	0.917 ± 0.103
MSCB Auxiliary	0.897 ± 0.120	0.8784 ± 0.123
TARNet	0.721 ± 0.129	0.666 ± 0.149
InceptionTime	0.789 ± 0.129	0.732 ± 0.150

Overall, the results are positive and support the hypothesis that deep neural networks designed for time series data can provide effective classification of extraneural ENG. Data augmentation has further been demonstrated to boost performance in this application.

## 4. Discussion

### 4.1 Signal length and application in closed-loop control devices

#### 4.1.1 Latency

When developing closed-loop neuroprosthetics for motor function, it is crucial to consider the latency of the feedback pathway. For comparison, in the myoelectric control literature, a delay of 100–125 ms has been found to be acceptable [38]. From Table 1, it can be seen that model performance did not differ greatly between signal lengths of 1500 and 2250, but a large drop in performance was seen when using signal lengths of 100. At a sampling rate of 30kHz, a signal length of 1500 corresponds to 50 ms. Furthermore, in this study, the average inference time for a single signal varied between 1 and 2.5ms, as shown in Table 4. This indicates that there is potential to integrate this model into a closed-loop control device. While the computational requirements of deep learning models may pose challenges for implantable systems, recent work suggests that CNNs for ENG classification can be reduced in size with only a minor impact on performance [39].

**Table 4:** Inference time for each model on 1500 samples run on a NVIDIA GEFORCE RTX 3090 GPU.

Algorithm	Inference Time (ms)
MSCB Single Classifier	1.584
MSCB Auxiliary	1.936
TARNet	2.064
InceptionTime	2.334

#### 4.1.2 Accuracy vs time resolution

From Table 1, it is clear that increasing the window size has a positive impact on the classification accuracy of time series models. This is consistent with previous finding applying classifiers to longer windows of data in this dataset [40]. One possible factor to explain the trend observed in the present study is the possibility of long windows containing multiple nCAPS, thereby allowing the model to learn features about the firing rate of each pathway and providing another basis for classifying signals. In contrast, a window size of 100 can only contain a single nCAP. However, it is important to analyze the trade-off to a longer window.

In this study, all signals only contained nCAPs from a single class. In real situations, this homogeneity in activity would not be present. This is not a significant problem for windows that are small enough to only contain a single nCAP but is a challenge with larger windows as nCAPs from multiple pathways may be observed in a single window.

Furthermore, a longer window decreases the time resolution of the method, leading to a slower time to detect changes in the activity of target pathways due to both the longer window needed and the longer processing time.

### 4.2 Model comparison

From Table 2 it is observed that overall, the MSCB networks had better performance than InceptionTime and TARNet. For InceptionTime, this may be attributed to the increased complexity of the network*¸* which uses 3 Inception Modules per Inception Block. This results in a much deeper network which can lead to the vanishing/exploding gradient problem [41]. While residual connections and layer normalization may help mitigate this problem, neither completely solve the issue.

TARNet’s performance was the worst among all networks tested. This result may stem from the low inductive bias of the transformer architecture. The low inductive bias of the transformer architecture is very beneficial in problems with large amounts of data as it allows the model to do a more global search for an optimal solution. However, with smaller datasets, inductive biases can provide a constraint on the search space to help the model find a good local optimum.

The performance difference between the MSCB Single Classifier and MSCB Auxiliary was minimal. This indicates that the addition of a residual connection did not significantly impact the performance of the network. Furthermore, adding the residual connection increases the size of the network and thus slows the speed of inferences. Therefore, we conclude that the MSCB Single Classifier model was the preferred approach among the four time-series architectures investigated.

### 4.3 Data augmentation and data limitations

**Fig 7:**
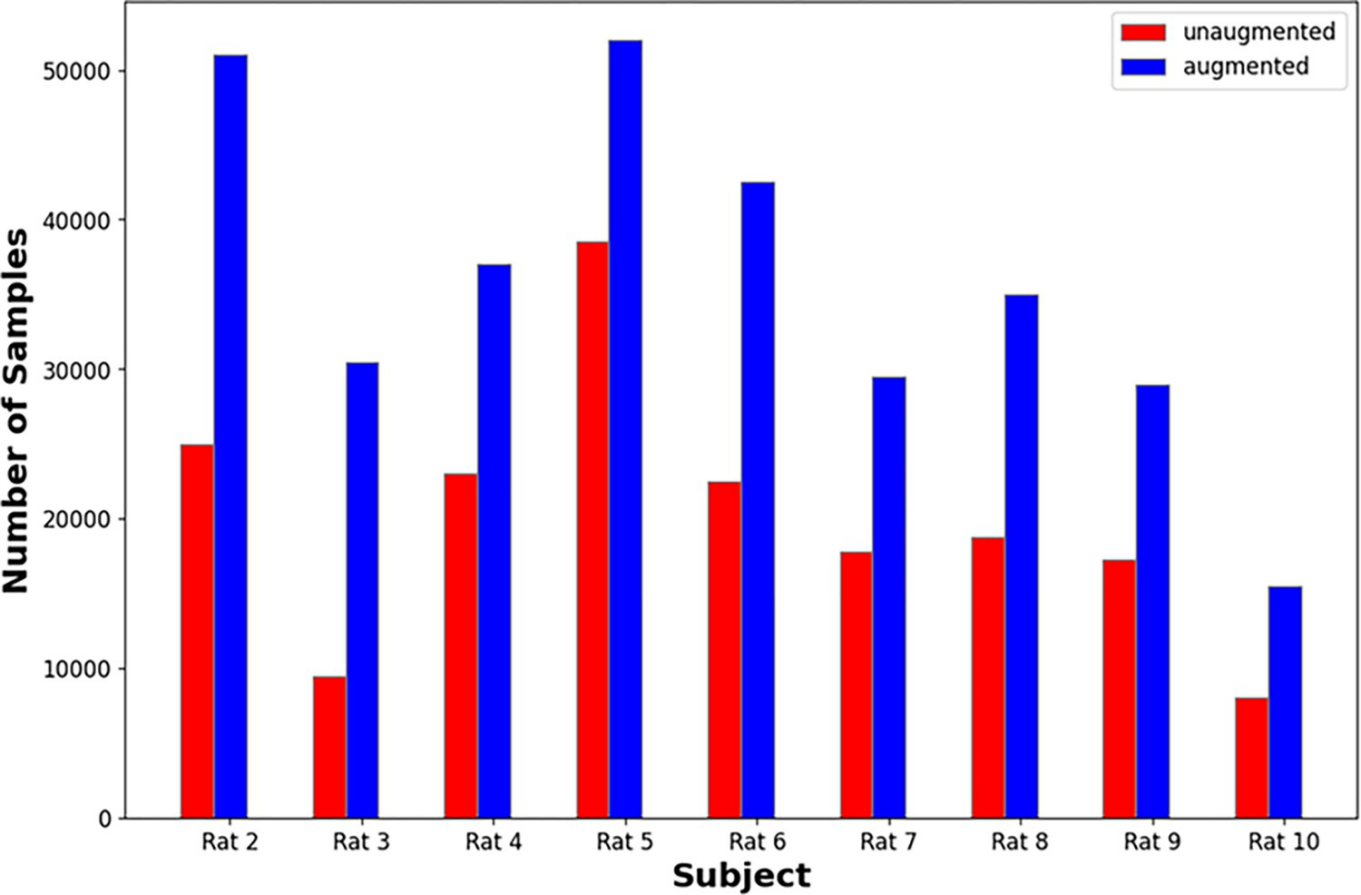
Size comparison of unaugmented and augmented datasets.

Comparing Tables 2 and 3, it can be seen that data augmentation improved performance for all networks. This is in line with expectations as the data-augmented dataset is over twice as large as the minimum dataset as shown below in Fig 7.

This finding validates the data augmentation techniques used; if data augmentation created invalid synthetic data, a drop in performance should have been observed.

Out of all models, TARNet had the biggest boost in performance from data augmentation. This is expected as the transformer require more data compared to CNNs. This result suggests that if more data can be collected a transformer model may achieve state of the art performance.

### 4.4 Future work

To reach the goal of integrating these models into closed-loop neuroprosthetic systems, there are more challenges to be overcome. First, models will need to be trained and evaluated on datasets that contain signals from multiple pathways at once. In this study, there was only a single stimulation occurring at one time. This includes evaluating model performance on a broader number of situations including more complex movement patterns which will yield more complex patterns of neural activity. The model should also be applied to different nerves as the number of fascicles will differ between nerves.

Second, the performance of the model should be evaluated in chronic applications to better understand the effect of issues that may occur during long-term implantation, such as damage or movement of the electrode and the growth of encapsulation tissue.

Third, models will need to be applied to signals that are collected from awake and moving subjects. At this time, all subjects were fully anesthetized which reduces the amount of noise from other pathways and minimizes any shifting of the nerve cuff. When collecting from awake subjects, there will likely be more signal artefacts present and increased temporal overlap of signals.

Fourth, it will be crucial to deploy models that are sufficiently resource-efficient to be integrated into implantable systems [42].

## 5 Conclusion

This work presents a novel method for classifying extraneural peripheral nerve signals. Using ENG recordings from a 56-contact nerve cuff electrode, we investigated several deep learning models designed for time-series classification. A multi-scale 1D-CNN combined with data augmentation strategies was found to provide very high classification accuracy, 0.936 ± 0.084, when applied to 50 ms windows. These results provide exciting new areas of investigation for the development of closed-loop neuroprosthetic systems.

## Supporting information

S1 File (DOCX)
