# Supplementary material for: Time series classification of multi-channel nerve cuff recordings using deep learning
Source: PLoS One. 2024 Mar 12;19(3):e0299271. doi: 10.1371/journal.pone.0299271 (PMC10931496; doi:10.1371/journal.pone.0299271)
Supplement: S1 File — (DOCX) [file pone.0299271.s001.docx]

**Supplementary Information S1 –** Training Hyperparameters and Settings

All models were trained with the following training hyperparameters with small modifications made if needed.

Table S1: Training Hyperparameters and Optimizer Information

| **Parameter** | **Value** |
| --- | --- |
| Learning rate | 0.00005 |
| Number of Epochs | 400 |
| Early Stopping Patience | 3 |
| Early Stopping Condition | Validation Accuracy > 85% |
| Batch Size | 16 |
| Optimizer | Adaptive Moment Estimation |

**Supplementary Information** S2 – MSCB Single Classifier Architecture

Table S2: MSCB Single Classifier Architecture Information

| **Layer** | **Layer Types** | **Kernel Size** | **Stride** | **Output # of Filters** |
| --- | --- | --- | --- | --- |
| **1a** | 1D Convolution | 1 | 1 | 64 |
| **1b** | 1D Convolution | 3 | 1 | 64 |
| **1c** | 1D Convolution | 5 | 1 | 64 |
| **1d** | 1D Maxpooling | 3 | 5 | 128 |
| **2a** | 1D Maxpooling | 1 | 1 | 64 |
| **2b** | 1D Maxpooling | 3 | 1 | 64 |
| **2c** | 1D Maxpooling | 5 | 1 | 64 |
| **2d** | 1D Convolution | 24 | 1 | 128 |
| **3** | Concatenation | - | - | 300 |
| **Main Pathway** | | | | |
| **4** | 1D Convolution | 112 | 1 | 64 |
| **5** | 1D Maxpooling | 3 | 5 | 64 |
|  | **Layer Type** | **# Nodes** | **Other Details** |  |
| **6** | Flatten | - | - |  |
| **7** | Fully Connected | 400 | - |  |
| **8** | Dropout | - | P=0.5 |  |
| **9** | Fully Connected | 1024 | - |  |
| **10** | Fully Connected | 3 | - |  |

**Supplementary Information S**3 – MSCB Auxiliary Architecture

**Table S3:** MSCB Auxiliary Architecture Information

| **Layer** | **Layer Type** | **Kernel Size** | **Stride** | **Output # of Filters** |
| --- | --- | --- | --- | --- |
| **1a** | 1D Convolution | 1 | 1 | 64 |
| **1b** | 1D Convolution | 3 | 1 | 64 |
| **1c** | 1D Convolution | 5 | 1 | 64 |
| **1d** | 1D Maxpooling | 3 | 5 | 128 |
| **2a** | 1D Maxpooling | 1 | 1 | 64 |
| **2b** | 1D Maxpooling | 3 | 1 | 64 |
| **2c** | 1D Maxpooling | 5 | 1 | 64 |
| **2d** | 1D Convolution | 24 | 1 | 128 |
| **3** | Concatenation | - | - | 300 |
| **Main Pathway** | | | | |
| **4** | 1D Convolution | 112 | 1 | 64 |
| **5** | 1D Maxpooling | 3 | 5 | 64 |
|  | **Layer Type** | **# Nodes** | **Other Details** |  |
| **6** | Flatten | - | - |  |
| **7** | Fully Connected | 400 | - |  |
| **8** | Dropout | - | P=0.5 |  |
| **9** | Fully Connected | 1024 | - |  |
| **10** | Fully Connected | 3 | - |  |
| **Auxiliary Pathway** | | | | |
|  | **Layer Type** | **Kernel Size** | **Stride** | **Output # of Filters** |
| **4** | MPOOL | 10 | 10 | 300 |
|  | **Layer Type** | **# Nodes** | **Other Details** |  |
| **5** | Flatten | - | - |  |
| **6** | Fully Connected | 400 | - |  |
| **7** | Dropout | - | P=0.5 |  |
| **8** | Fully Connected | 1024 | - |  |
| **9** | Fully Connected | 3 | - |  |

**Supplementary Information** S4 – InceptionTime Parallel Convolutional Block Architecture

**Table S4:** InceptionTime Parallel Convolutional Block Architecture Information

| **Parallel Convolutions** | | | | |
| --- | --- | --- | --- | --- |
| **Layer** | **Layer Type** | **Kernel Size** | **Stride** | **Output # of Filters** |
| **P1a** | 1D Convolution | 1 | 1 | 32 |
| **P1b** | 1D Maxpooling | 3 | 1 | 32 |
| **P2a** | 1D Convolution | 9 | 1 | 32 |
| **P2b** | 1D Convolution | 19 | 1 | 32 |
| **P2c** | 1D Convolution | 39 | 1 | 32 |
| **P2d** | 1D Convolution | 1 | 1 | 32 |
| **P3** | Concatenation | - | - | 128 |
| **P4** | Batch Norm | - | - | 128 |

**Supplementary Information** S5 – InceptionTime Parallel Convolutional Block Architecture

**Table S5:** InceptionTime Architecture Information

| **Layer** | **Layer Type** | **Kernel Size** | **Stride** | **Output # of Filters** |
| --- | --- | --- | --- | --- |
| **P** | Inception Block | - | - | 128 |
| **R1** | 1D Convolution | 1 | 1 | 128 |
| **R2** | Batch Norm | - | - | 128 |
| **2** | Residual | **Note:** Layer R2 + Layer P | | |
| **3** | Averagepooling | - | - | 12 |
|  | **Layer Type** | **# Nodes** | **Other Details** |  |
| **4** | Flatten | - | - |  |
| **5** | Fully Connected | 1536 | - |  |
| **6** | Fully Connected | 3 | - |  |

**Supplementary Information** S6 – TARNet Architecture

**Table S6:** TARNet Architecture Information

| **Pre-Attention** | | | | |
| --- | --- | --- | --- | --- |
| **Layer** | **Layer Type** | **# Nodes** | **Other Details** |  |
| **1** | Fully Connected | 512 | 1 |  |
| **2** | Layer Normalization |  |  |  |
| **3** | Layer Normalization |  |  |  |
| **M** | Multi-Head Attention | 2048 | Heads = 8 |  |
| **5** | Dropout | - | P = 0.1 |  |
| **6** | Layer Normalization | 128 |  |  |
| **7** | Fully Connected |  |  |  |
| **8** | Dropout | - | P = 0.3 |  |
| **9** | Layer Normalization |  |  |  |
| **10** | Fully Connected | 128 |  |  |
| **11** | Dropout | - | P = 0.3 |  |
| **12** | Fully Connected | 128 |  |  |
| **13** | Dropout | - | P = 0.3 |  |
| **14** | Fully Connected | 128 |  |  |
| **15** | Cross Entropy Loss |  |  |  |
| **16** | Prediction |  |  |  |
